# *In Situ* Preparation of Biomimetic Thin Films and Their Surface-Shielding Effect for Organisms in High Vacuum

**DOI:** 10.1371/journal.pone.0078563

**Published:** 2013-11-13

**Authors:** Hiroshi Suzuki, Yasuharu Takaku, Isao Ohta, Daisuke Ishii, Yoshinori Muranaka, Masatsugu Shimomura, Takahiko Hariyama

**Affiliations:** 1 Departments of Chemistry, Hamamatsu University School of Medicine, Higashi-ku, Hamamatsu, Japan; 2 Departments of Biology, Hamamatsu University School of Medicine, Higashi-ku, Hamamatsu, Japan; 3 Research Equipment Center, Hamamatsu University School of Medicine, Higashi-ku, Hamamatsu, Japan; 4 Center for Fostering Young and Innovative Researchers, Nagoya Institute of Technology, Gokiso-cho, Showa-ku, Nagoya, Japan; 5 World Premier International−Advanced Institute for Materials Research, Tohoku University, Aoba-ku, Sendai, Japan; 6 Institute of Multidisciplinary Research for Advanced Materials, Tohoku University, Aoba-ku, Sendai, Japan; 7 CREST, Japan Science and Technology Agency, Kawaguchi, Japan; University of Akron, United States of America

## Abstract

Self-standing biocompatible films have yet to be prepared by physical or chemical vapor deposition assisted by plasma polymerization because gaseous monomers have thus far been used to create only polymer membranes. Using a nongaseous monomer, we previously found a simple fabrication method for a free-standing thin film prepared from solution by plasma polymerization, and a nano-suit made by polyoxyethylene (20) sorbitan monolaurate can render multicellular organisms highly tolerant to high vacuum. Here we report thin films prepared by plasma polymerization from various monomer solutions. The films had a flat surface at the irradiated site and were similar to films produced by vapor deposition of gaseous monomers. However, they also exhibited unique characteristics, such as a pinhole-free surface, transparency, solvent stability, flexibility, and a unique out-of-plane molecular density gradient from the irradiated to the unirradiated surface of the film. Additionally, covering mosquito larvae with the films protected the shape of the organism and kept them alive under the high vacuum conditions in a field emission-scanning electron microscope. Our method will be useful for numerous applications, particularly in the biological sciences.

## Introduction

Biocompatible functional films are becoming increasingly important for bioengineering. The unique properties of biocompatible films, which are fabricated from biocompatible molecules with hydrophilic groups, have been exploited in engineering and bioengineering [1]–[3]. Water-insoluble self-standing polymer films have been the subject of intensive research for many applications. Physical vapor deposition (PVD) and chemical vapor deposition (CVD) combined with plasma polymerization are well-established methods for making self-standing films from small molecules [4], [5]. Plasma generated during the deposition process generates radicals with sufficient energy to allow the mutual binding of small molecules, and thus initiates polymerization [6]–[9]. However, during PVD plasma polymerization, the small molecules must be in the gaseous state, and in CVD they must be carried by a gaseous plasma source. Self-standing biocompatible films have yet to be prepared by PVD or CVD because the monomers for biocompatible polymers are nonvolatile.

Frank-Finney *et al.* reported that a self-standing film was formed on an ionic liquid in vacuum by CVD [10]. However, nonvolatile compounds were used as the substrate for the self-standing films. In contrast, we have recently reported that self-standing films can be fabricated directly from surfactant monomers dissolved in a volatile liquid by a simple one-step polymerization [11]. Covering the living larvae of *Drosophila* with a layer of extra cellular substance (ECS) polymerized by plasma or electron beam irradiation made the organisms highly tolerant to high vacuum. Furthermore, a 1% aqueous solution of the surfactant polyoxyethylene (20) sorbitan monolaurate (TW 20) formed a thin film which mimicked the ECS [11]. In this study, we investigated biomimetic ECS films made by plasma polymerization from surfactants, water-soluble polymers, monosaccharides, polysaccharides, lipids, amino acids, and ionic liquids. These films showed both typical polymer properties and a unique out-of-plane molecular density gradient from the irradiated to the unirradiated film surface. We demonstrated that the biomimetic ECS films can protect the shape of organisms and keep them alive in the high vacuum of a field-emission scanning electron microscope (FE-SEM).

## Materials and Methods

### Materials

All monomers used in this study (Table 1 and Fig. S1) were of reagent grade and were purchased from Wako Pure Chemical Co., Tokyo Kasei Kogyo Co., Sigma-Aldrich Japan Co., Kanto Chemical Co., or Dojindo Co. in Japan.

**Table 1:** Self-standing films fabricated by plasma polymerization.

Sample	Monomer	Polymerization site	Solvent	State*
**1**	Tween 20^**^^a)^	PEO chain	Water	o○
**2**	Tween 40 ^a)^	PEO chain	Water	o○
**3**	Tween 60 ^b)^	PEO chain	Water	o○
**4**	Tween 80 ^a)^	PEO chain	Water	o○
**5**	Brij 35^** a)^	PEO chain	Water	o○
**6**	Triton X-100^**^^c)^	PEO chain	Water	o○
**7**	Poly (ethylene oxide) ^c)^	PEO chain	Water	o○
**8**	Pluronic F-127^**^^c)^	PEO chain	Water	o○
**9**	Pluronic F-68 ^c)^	PEO chain	Water	o○
**10**	Lecithin (from soy bean) ^d)^	Multiple OH	Ethanol	o○
**11**	Tannic acid ^c)^	Multiple OH	Ethanol	o○
**12**	Tetraethoxysilane ^b)^	Multiple OH	Ethanol	o○
**13**	Span 20 ^b)^	Multiple OH	Ethanol	o○
**14**	D-Maltose ^b)^	Multiple OH	Water	o○
**15**	Trehalose C12 ^e)^	Multiple OH	Water	o○
**16**	D-Glucose ^a)^	Multiple OH	Water	o○
**17**	n-Dodecyl-β-D-maltoside^**^^e)^	Multiple OH	Water	o○
**18**	MEGA-8^**^^e)^	Multiple OH	Water	○
**19**	CHAPS^**^^e)^	Multiple OH	Water	○
**20**	D-Trehalose ^b)^	Multiple OH	Water	○
**21**	Sodium cholate^**^^e)^	Multiple OH	Water	○
**22**	n-Octyl-β-D-glucoside^**^^e)^	Multiple OH	Water	○
**23**	Inulin ^b)^	Multiple OH	Water	△
**24**	Pullulan ^b)^	Multiple OH	Water	△
**25**	D-Sorbitol ^b)^	Multiple OH	Water	△
**26**	L-Tyrosine ^a)^	Multiple OH	Water	△
**27**	L-Glutamic acid ^a)^	Multiple OH	Water	△
**28**	L-Aspartic acid ^a)^	Multiple OH	Water	△
**29**	Lauric acid ^b)^	Single OH	Ethanol	○
**30**	Stearic acid n-dodecyl ester ^b)^	Single OH	Ethanol	○
**31**	Docosanoic acid ^b)^	Single OH	Ethanol	○
**32**	L-Proline ^a)^	Single OH	Water	△
**33**	L- Lysine ^a)^	Single OH	Water	△
**34**	L-Histidine ^a)^	Single OH	Water	△
**35**	Linolenic acid ^b)^	OH & C = C double bond	Ethanol	o○
**36**	Linoleic acid ^b)^	OH & C = C double bond	Ethanol	o○
**37**	Oleic acid ^b)^	OH & C = C double bond	Ethanol	o○
**38**	Erucic acid ^b)^	OH & C = C double bond	Ethanol	o○
**39**	Methacroylcholine chloride ^b)^	OH & C = C double bond	Ethanol	o○
**40**	L-Glutamine ^a)^	OH & C = C double bond	Water	○
**41**	L-Arginine ^a)^	OH & C = N double bond	Water	○
**42**	1,3-Diallylimidazolium bromide ^d)^	C = C double bond	Ethanol	o○
**43**	1,3-Diallylimidazolium tetrafluoroborate ^d)^	C = C double bond	Ethanol	o○
**44**	1,3-Diallylimidazolium bis (trifluoromethanesulfonyl) imide ^d)^	C = C double bond	Ethanol	o○
**45**	1-Buthyl-3-methylimidazolium tetrafluoroborate ^d)^	C = C double bond	Ethanol	○
**46**	1-Butyl-3-methylimidazolium bis (trifluoromethanesulfonyl) imide ^b)^	C = C double bond	Ethanol	○

* o○: Large, thick, stable film ○: Small, thin, stable film, △: Unstable film ** SEM observation of living organisms

a) Wako Pure Chemical Co., b) Tokyo Kasei Kogyo Co., c) Sigma-Aldrich Japan Co., d) Kanto Chemical Co., e) Dojindo Co.

### Preparation of insoluble plasma-irradiated films

**Figure 1:**
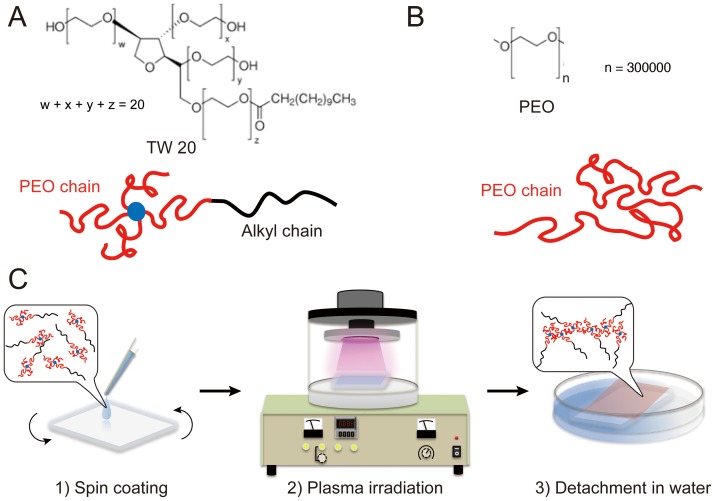
Preparation of free-standing films by plasma irradiation. Chemical structure and schematic model of (A) TW 20 and (B) PEO. (C) Schematic illustration of the preparation method. A water-soluble layer spin-coated on a glass substrate becomes a water-insoluble film upon plasma irradiation.

An aqueous or ethanol solution of 50 wt % TW 20 (0.3 mL) was spin-coated on a 25×25 mm glass plate at 3000 rpm for 5 s using a spin-coater (SC8001, Aiden). The liquid TW 20 layer was placed in a standard ion-sputtering device (MSP-20-UM, Vacuum Device). The metal plate in the device was removed so the plasma ions were produced from the residual air molecules. The liquid layer was irradiated with plasma for 10 min at 1.0 kV DC (30.0 mA) under a vacuum of ∼1.0 Pa at room temperature. The irradiated glass plate was immersed in distilled water to separate the water-insoluble film from the glass plate. Water-insoluble films of poly (ethylene oxide) (PEO; average M_w_  = 300,000) or other monomers (Table 1) were prepared according to the same method. Figure 1C shows the preparation method for the self-standing films.

### Physical measurements

The thermal decomposition temperatures of the soluble monomers and the insoluble plasma-irradiated films were investigated with a thermogravimetric analyzer (TG/DTA-320, Seiko). The four samples (∼5.0 mg), which were the TW 20 or PEO monomers before plasma-irradiation and the corresponding films after plasma irradiation, were put in an aluminum vessel and dried under ambient conditions. The decrease in the mass of the sample was measured at 1 s intervals while the temperature was increased from 50 to 500°C at a rate of 10°C min^−1^ under a nitrogen atmosphere (200 mL min^−1^). The attenuated total reflectance Fourier transform infrared spectroscopy (ATR-FTIR) spectra were obtained with a FTIR spectrophotometer (FT/IR-6100, Jasco) equipped with an ATR holder (ATR PRO470-H, Jasco). The liquid monomer layers of TW 20 or PEO before plasma irradiation and the corresponding free-standing films after plasma irradiation were measured with a scan resolution of 2 cm^−1^.

### Surface features of plasma-irradiated films

The irradiated and unirradiated surface ultrastructures of the plasma-irradiated TW 20 film were measured by atomic force microscopy (AFM) using a scanning probe microscope (JSPM 5200, JEOL) under atmospheric pressure at room temperature (25°C). The AFM was used in AC mode at a scanning rate of 2.0 Hz and a scanning size of 2500×2500 nm.

### TEM observations of plasma irradiated films

The plasma-irradiated TW 20 films were immersed in an epoxy resin (Quetol 651, Nisshin EM, Japan) at room temperature for 4 h. The films were embedded in Quetol 651 and were allowed to polymerize for 48 h at 60°C. A vertical ultrathin section (150 nm) was cut using an ultramicrotome (Ultracut OmU4, Reichert-Jung). The specimens were mounted on nickel grids and stained with 2% OsO_4_ in distilled water for 10 min, and coated with carbon. Observations were performed using an electron microscope (JEM-1220, JEOL) at an accelerating voltage of 120 kV.

### SEM observations of organisms covered by biomimetic ECS films

SEM observations of living organisms were performed as described by Takaku *et al.* (2013). Briefly, fourth instar larvae of the mosquito *Aedes albopictus*, which have a soft cuticle not covered by ECS, were used. The larvae were collected from puddles and kept in the same water in which they were found. In order to exclude any effects from the water, they were transferred to distilled water at 24±1°C for two days prior to the experiment, with distilled water changes every 12 h. The larvae were rinsed thoroughly in distilled water 1 h before the experiment began. The animals were dipped in a 1% (v/v) monomer solution in distilled water for 1 min, blotted briefly on a dry filter paper to remove excess solution, and then irradiated with plasma to fabricate the plasma-treated films. The sample was placed in the SEM directly with no traditional treatment such as chemical fixation, dehydration, or ultrathin coating of electrically conducting materials.

## Results and Discussion

**Figure 2:**
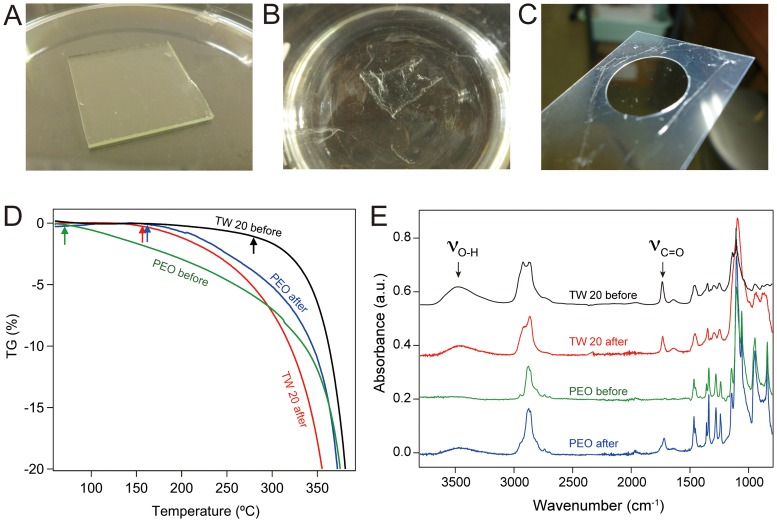
Physical measurements of the free-standing insoluble TW 20 film. Digital photographs of (A) the plasma-irradiated film, (B) the free-standing film removed from glass substrate and (C) the self-standing film after natural drying. (D) Thermogravimetric curves, and (E) ATR-FTIR spectra of TW 20 and PEO before and after plasma polymerization. Arrows in the thermogravimetric curves indicate the decomposition temperatures.

Figure 1C shows the preparation of the biomimetic ECS films for conventional structural analysis. The monomer layer was spin-coated on a glass substrate, and then placed in an irradiation chamber. The water-soluble layer, which consisted of the monomer and water, remained wet even under vacuum (10^−3^ Torr). Thin films were formed on the outermost surface of the monomer layer after plasma irradiation (Fig. 2A). The unreacted monomer was removed by immersing the plasma-irradiated films in a large volume of pure water and ethanol (Fig. 2B, Movie S1). The insoluble plasma-irradiated films were transferred to a substrate and allowed to dry under ambient conditions, demonstrating they were free standing (Fig. 2C). The thickness of the insoluble film was controlled by adjusting the electrical potential, the irradiation time, the distance to the irradiated surface, the concentration of the solution, and the spin-coating conditions.

The plasma polymerization was investigated with free-standing TW 20 or PEO films. PEO is a water-soluble polymer with the same structure as the hydrophilic portion of TW 20 (Fig. 1A and B). The thermodynamic properties of the monomers before plasma irradiation and the insoluble free-standing films after plasma irradiation were determined by thermogravimetric analysis (Fig. 2D). The decomposition temperatures of TW 20 and PEO before plasma irradiation were 380 and 70°C, respectively, whereas the insoluble TW 20 and PEO films after plasma irradiation decomposed above 160°C. This indicates that the insolubility of the free-standing TW 20 and PEO films after plasma irradiation was the driving force for the formation of the intermolecular ethoxy chains by plasma cross-linking. Figure 2E shows ATR-FTIR spectra of the surfaces before and after irradiation. The spectra before and after irradiation were similar, indicating that the chemical structures of TW 20 and PEO were not degraded by plasma irradiation. Slight differences in the hydroxyl stretching vibration at 3600–3400 cm^−1^ and in the carboxyl stretching vibration at 1745–1720 cm^−1^ were observed for the plasma-irradiated PEO film. The hydroxyl and carboxyl groups were generated on the plasma-irradiated surface of the water-insoluble PEO film, indicating that plasma irradiation caused the intermolecular cross-linking of the PEO chain and surface oxidation.

Thin sections stained with toluidine blue revealed that the thickness of the free-standing TW 20 film was approximately 10 µm. The staining was localized strongly at the plasma-irradiated surface (Fig. S2), because toluidine blue has a positive charge and binds selectively to negatively charged sites. It has been proposed that because air-derived plasma contains oxygen, negatively charged carboxyl and hydroxyl groups are localized on the irradiated surface. This was confirmed by X-ray photoelectron spectroscopy (XPS; Fig. S3), suggesting that the intense toluidine blue staining on the irradiated surface was caused by the generation of carboxyl groups by surface oxidation.

**Figure 3:**
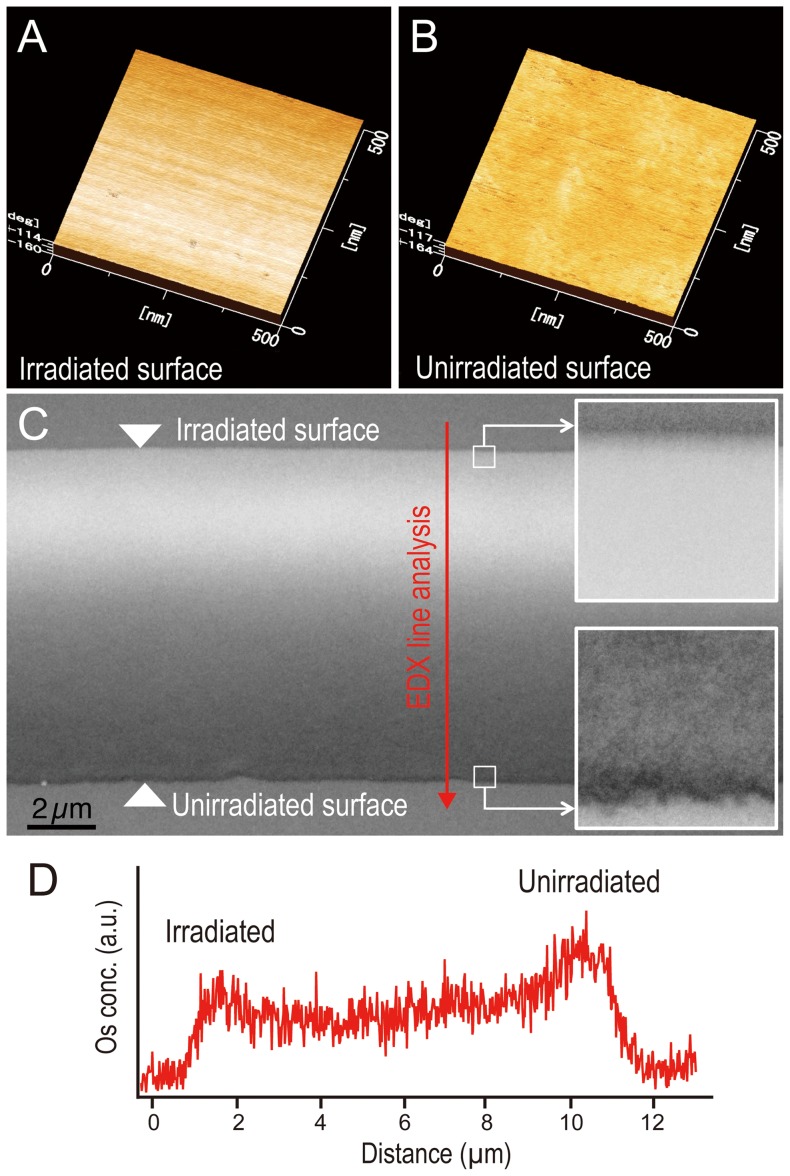
Morphological features of the TW 20 plasma-polymerized film. AFM images of the (A) plasma irradiated and (B) unirradiated surfaces, and (C) the TEM cross-sectional image. (D) Osmium concentration between the irradiated and unirradiated surfaces measured by EDX.

The free-standing films exhibited other typical properties, such as pH stability, transparency, a pinhole-free surface, and flexibility. The film was insoluble in solutions across a pH range of 3–10, although it dissolved in strong acids or bases, because the covalent bonds of the PEO chains decomposed. The transparency of the TW 20 film was greater than 95%. In addition, these films exhibited a unique structural feature. The AFM images show that the irradiated surface of the TW 20 film was uniform and smooth (Fig. 3A), whereas the unirradiated surface of the film was rough (Fig. 3B). The TEM images confirmed these morphological features, and also revealed an electron density gradient from the irradiated to the unirradiated surface (Fig. 3C). Energy dispersive X-ray (EDX) spectroscopy also showed an osmium concentration gradient between the irradiated and the unirradiated surfaces (Fig. 3D). This may be because the OsO_4_ stain, which binds strongly to the low-density polymerized domains, accumulated toward the unirradiated surface of the film. The difference was enhanced by the extent of the polymerization, particularly the cross-linking of the PEO chains. Thus, the free-standing thin films showed an out-of-plane molecular density gradient from the irradiated surface to the unirradiated surface. The fabrication of free-standing films has so far remained complex and challenging. Most methods involve the secondary processing of polymers, such as layer-by-layer (LBL) absorption of polyelectrolytes [12], [13], or filtration of nanofibrous materials [14]–[16]. Our free-standing thin films were formed by a simple one-step polymerization, and showed both typical polymer properties and a unique out-of-plane molecular density gradient from the irradiated to the unirradiated film surface.

Table 1 summarizes the free-standing films prepared from precursors with PEO chains (1–9); multiple hydrophilic groups (10–28); a single hydrophilic group (29–34); several double bonds (35–41); and an imidazolium ring typical of ionic liquids (42–46). All the free-standing films were insoluble in both water and ethanol. These results suggest that the radicals generated by the plasma promoted polymerization of both the common polymerizable functional groups, such as alkenes, and the hydrophilic groups, such as PEO chains, carboxyl, and hydroxyl groups, on the irradiated surface.

**Figure 4:**
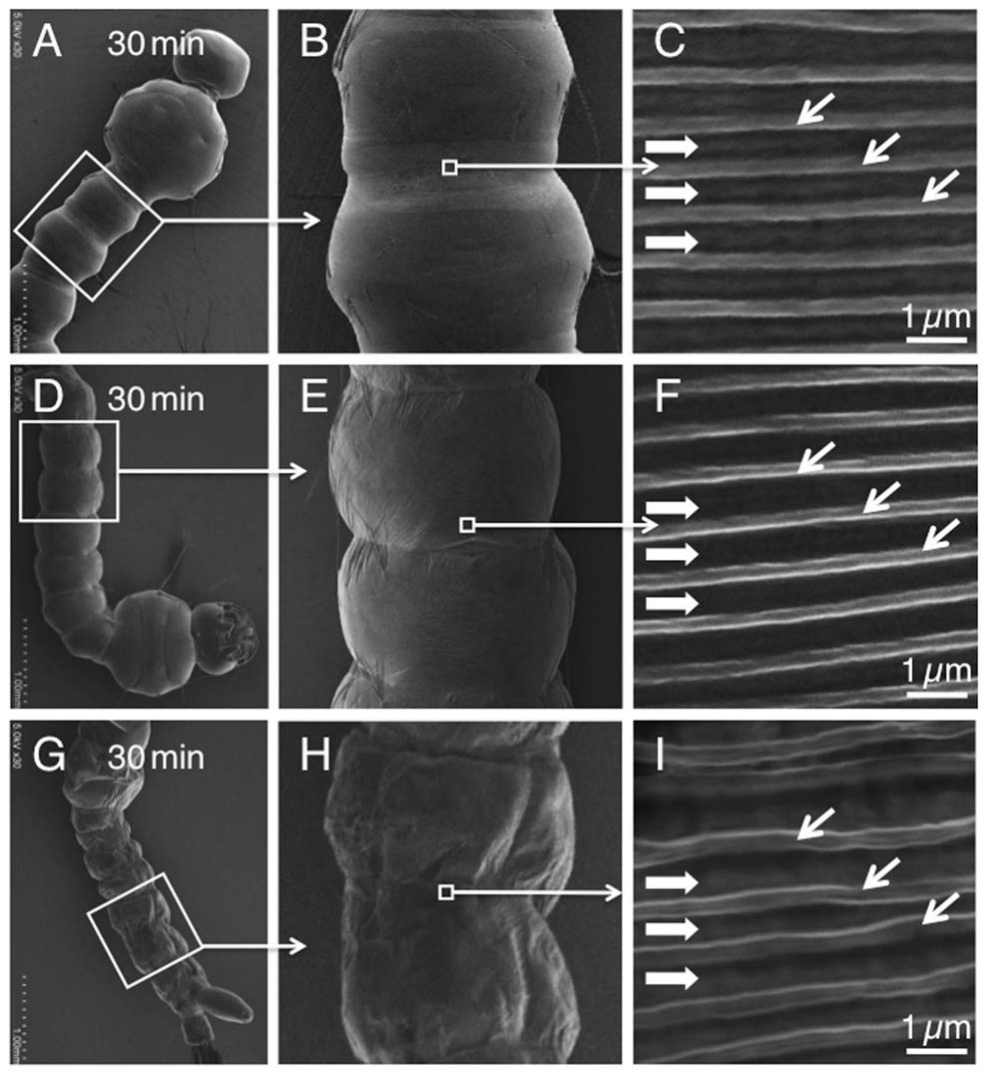
Typical FE-SEM images of the larvae protected by biomimetic ECS films and exposed to high vacuum for 30 min. The films were fabricated from Triton X-100 (A–C), pluronic F-127 (D–F), *n*-dodecyl-β-D-maltoside (G–I), respectively. Diagonal arrows indicate natural ridges; horizontal arrows indicate natural furrows on the surface of the animal, in C, F and I.

To investigate the surface-shielding effect of the films for organisms in high vacuum, we fabricated various biomimetic ECS films on the surface of mosquito larvae (*A. albopictus*). These organisms normally collapse and die from rapid dehydration under high vacuum conditions (10^−5^–10^−7^ Pa) in an FE-SEM [11]. The films made the larvae highly tolerant to the high vacuum (Fig. 4). In addition, as in the case of TW 20 [11], high-resolution images of the film-coated living organisms were obtained which showed the fine structures remained undamaged. Our method should facilitate the use of biomimetic ECS films with excellent hermetic properties, and should be suitable for numerous applications, especially in the biological sciences.

### Conclusion

Plasma-irradiated films prepared from various monomers showed both typical polymer properties and a unique out-of-plane molecular concentration gradient from the irradiated to the unirradiated surface. The films functioned as biomimetic ECS films, keeping mosquito larvae alive under high vacuum conditions and maintaining the shape of the organisms. This simple thin-film fabrication method should be suitable for producing more sophisticated materials, and has the potential to initiate new areas of research in physics, chemistry, and biology.

## Supporting Information

Figure S1 **Chemical structures of various monomers.** Structures of selected monomers used for film fabrication. Compound numbers correspond to those in Table 1.
(TIF)

Figure S2 **A cross-sectional light micrograph of the TW 20 film stained with toluidine blue.** The staining was localized at the plasma-irradiated surface.
(TIF)

Figure S3 **Surface oxidation of biomimetic ECS films prepared by plasma irradiation.** XPS spectra of (A) the irradiated surface and (B) the unirradiated surface of the TW 20 film. Arrows in the spectrum of the irradiated surface indicate the presence of carboxyl groups.
(TIF)

Movie S1 **TW 20 film in an aqueous solution.** The self-standing plasma-irradiated film shows the property of water-insoluble. The film has the flexibility, when it is pipetted in the water.
(MP4)

Movie S2 **TW 20 film shielding on the vial.** The plasma-irradiated film shows the excellent hermetic property.
(MP4)
