# Supplementary figures and images for: In Situ Preparation of Biomimetic Thin Films and Their Surface-Shielding Effect for Organisms in High Vacuum
Source: PLoS One. 2013 Nov 13;8(11):e78563. doi: 10.1371/journal.pone.0078563 (PMC3827240; doi:10.1371/journal.pone.0078563)

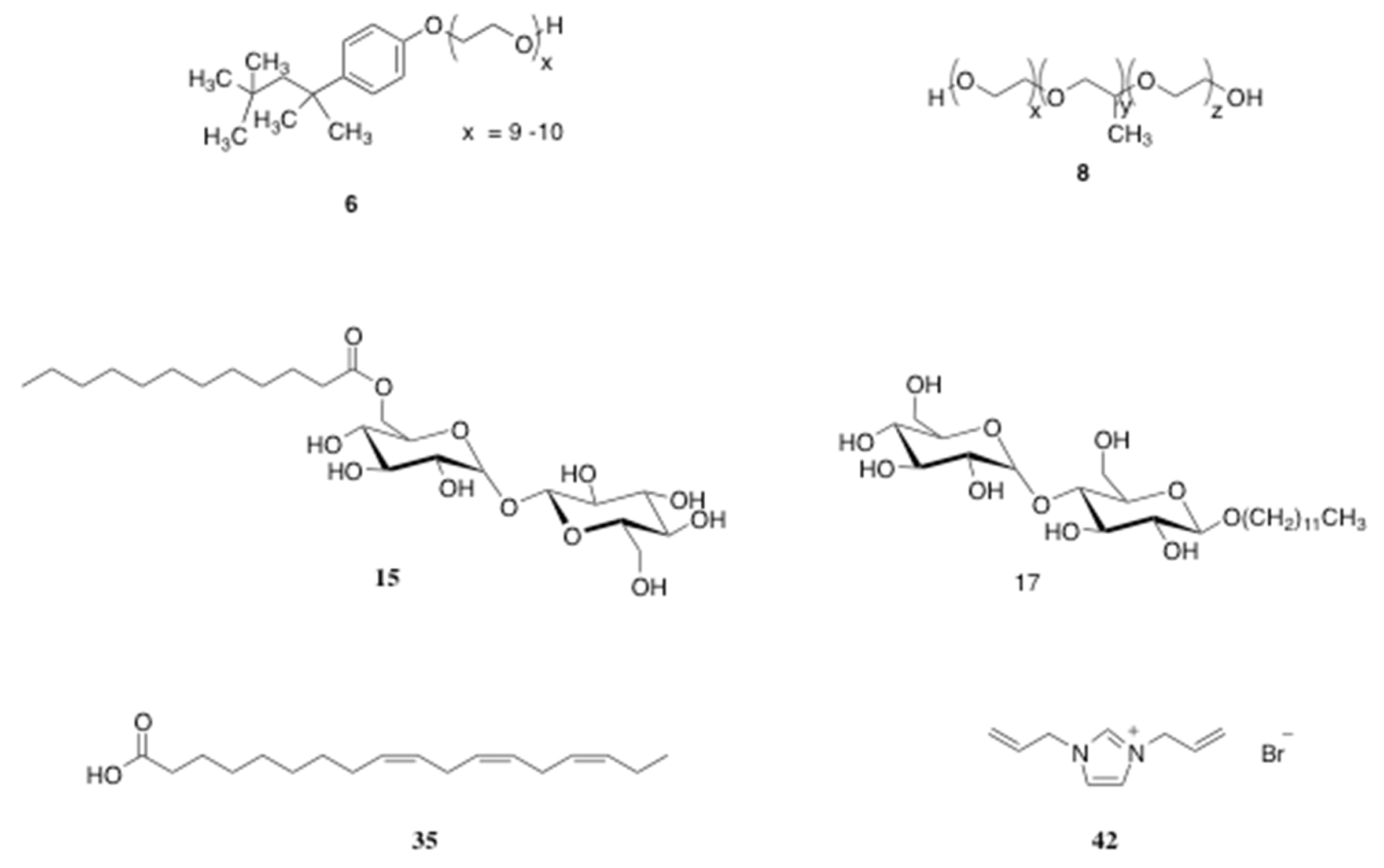

Supplement: Figure S1 — Chemical structures of various monomers. Structures of selected monomers used for film fabrication. Compound numbers correspond to those in Table 1. (TIF) [file pone.0078563.s001.tif]

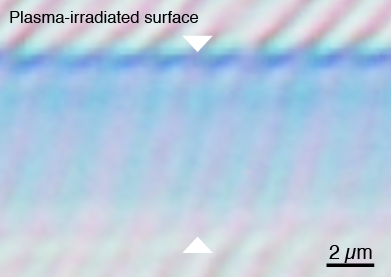

Supplement: Figure S2 — A cross-sectional light micrograph of the TW 20 film stained with toluidine blue. The staining was localized at the plasma-irradiated surface. (TIF) [file pone.0078563.s002.tif]

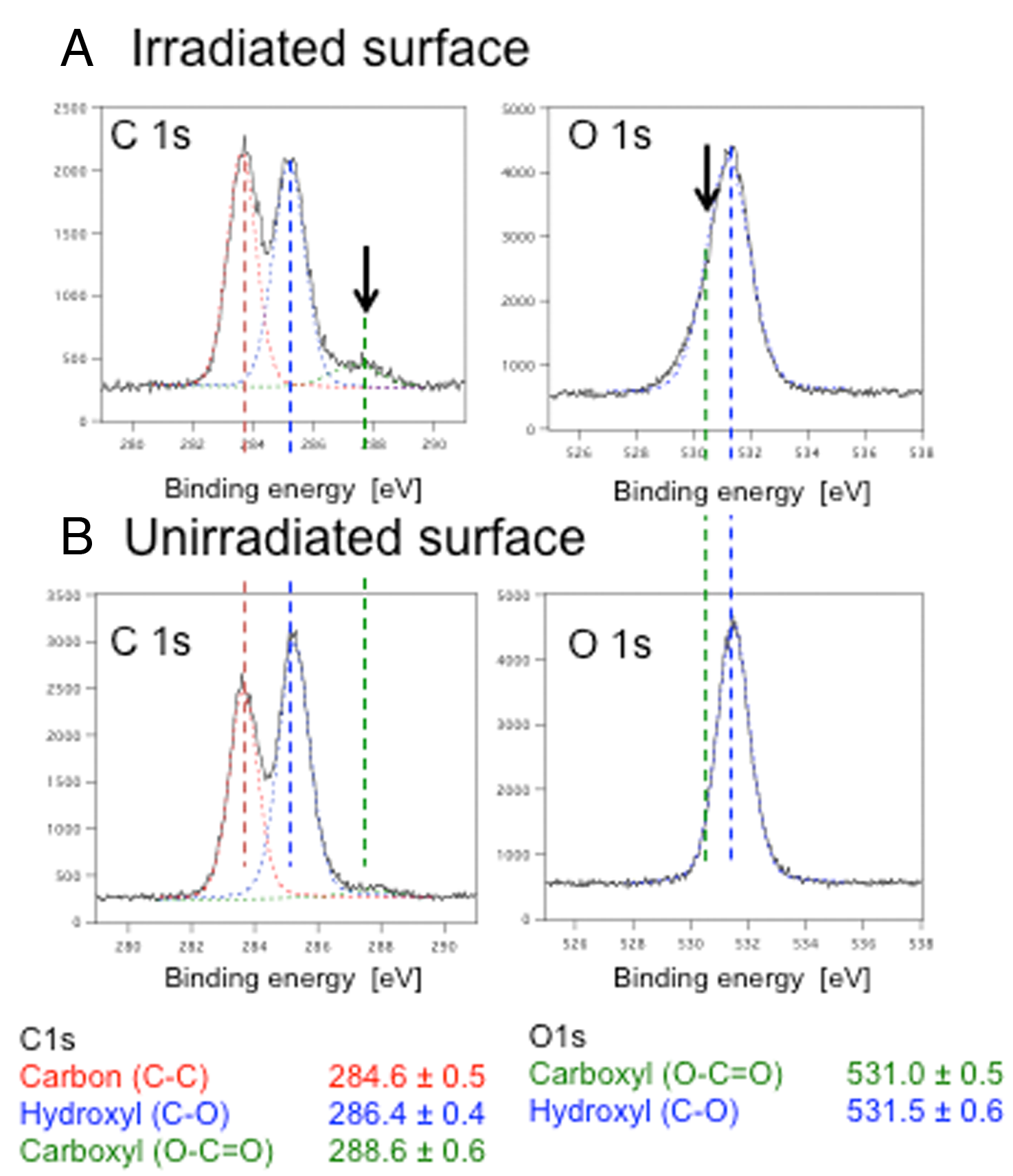

Supplement: Figure S3 — Surface oxidation of biomimetic ECS films prepared by plasma irradiation. XPS spectra of (A) the irradiated surface and (B) the unirradiated surface of the TW 20 film. Arrows in the spectrum of the irradiated surface indicate the presence of carboxyl groups. (TIF) [file pone.0078563.s003.tif]
